# Plant Hormone Salicylic Acid Produced by a Malaria Parasite Controls Host Immunity and Cerebral Malaria Outcome

**DOI:** 10.1371/journal.pone.0140559

**Published:** 2015-10-14

**Authors:** Ryuma Matsubara, Hiroka Aonuma, Mikiko Kojima, Michiru Tahara, Syed Bilal Ahmad Andrabi, Hitoshi Sakakibara, Kisaburo Nagamune

**Affiliations:** 1 Department of Parasitology, National Institute of Infectious Diseases, Shinjyuku, Tokyo, Japan; 2 Graduate School of Life and Environmental Sciences, University of Tsukuba, Tsukuba, Ibaraki, Japan; 3 Center for Sustainable Resource Science, RIKEN, Yokohama, Kanagawa, Japan; 4 Faculty of Life and Environmental Sciences, University of Tsukuba, Tsukuba, Ibaraki, Japan; Université Pierre et Marie Curie, FRANCE

## Abstract

The apicomplexan parasite *Toxoplasma gondii* produces the plant hormone abscisic acid, but it is unclear if phytohormones are produced by the malaria parasite *Plasmodium* spp., the most important parasite of this phylum. Here, we report detection of salicylic acid, an immune-related phytohormone of land plants, in *P*. *berghei* ANKA and *T*. *gondii* cell lysates. However, addition of salicylic acid to *P*. *falciparum* and *T*. *gondii* culture had no effect. We transfected *P*. *falciparum* 3D7 with the *nahG* gene, which encodes a salicylic acid-degrading enzyme isolated from plant-infecting *Pseudomonas* sp., and established a salicylic acid-deficient mutant. The mutant had a significantly decreased concentration of parasite-synthesized prostaglandin E_2_, which potentially modulates host immunity as an adaptive evolution of *Plasmodium* spp. To investigate the function of salicylic acid and prostaglandin E_2_ on host immunity, we established *P*. *berghei* ANKA mutants expressing *nahG*. C57BL/6 mice infected with *nahG* transfectants developed enhanced cerebral malaria, as assessed by Evans blue leakage and brain histological observation. The *nahG*-transfectant also significantly increased the mortality rate of mice. Prostaglandin E_2_ reduced the brain symptoms by induction of T helper-2 cytokines. As expected, T helper-1 cytokines including interferon-γ and interleukin-2 were significantly elevated by infection with the *nahG* transfectant. Thus, salicylic acid of *Plasmodium* spp. may be a new pathogenic factor of this threatening parasite and may modulate immune function via parasite-produced prostaglandin E_2_.

## Introduction

Malaria is a dominant human infectious disease that affects more than 200 million people and caused 627,000 deaths in 2012 [1]. *Plasmodium* is an apicomplexan parasitic protist that causes malaria. Apicomplexan parasites are thought to originate from the second endosymbiosis of ancestral red algae [2]. All apicomplexan parasites except *Cryptosporidium* and some Gregarine possess a plastid, apicoplast, which is thought to be derived from an ancient endosymbiont [2]. Therefore, apicomplexans have conserved plant-like signaling mechanisms. We previously reported that *Toxoplasma gondii*, an apicomplexan parasite, produces a plant hormone, abscisic acid (ABA) [3]. ABA promotes the calcium-dependent egress of parasites from the host cell, and inhibition of the biosynthesis of ABA leads the parasite to differentiate into a chronic infection cycle. This hormone is a key molecule that determines parasite pathogenicity, and the presence of a plastid suggests that other phytohormones might be found in apicomplexans. *Plasmodium* spp. is also likely to have conserved phytohormone signaling that may contribute to pathogenicity. In this study, we examined phytohormones of malaria parasites as potential pathogenic factors, and identified salicylic acid (SA) as a phytohormone in *T*. *gondii* and *Plasmodium* spp. cell lysates.

In higher plants, SA regulates the pathogen-resistance system through a mechanism referred to as systemic acquired resistance [4]. Pathogen-induced SA is delivered to the whole plant to upregulate expression of pathogenesis-related (PR) genes [4]. Recently, the receptors for SA were identified in *Arabidopsis thaliana* and named as nonexpresser of PR genes (NPR) 3 and 4 [5]. NPR3 and 4 are adaptors of Cullin 3 ubiquitin E3 ligase, which degrades the transcription cofactor, NPR1. SA is synthesized by two pathways: a major benzoic acid (BA) pathway, in which L-phenylalanine is converted to SA by phenylalanine ammonia lyase and BA 2-hydroxylase [6]; and a minor isochorismate synthase (ICS) pathway [7], which is thought to be dominant in *A*. *thaliana* and requires ICS as the key enzyme.

Metazoans do not synthesize SA, but SA has a strong activity in animals. SA and its acetylated derivative, acetylsalicylic acid, inhibit prostaglandin (PG) synthesis in animals and are used as non-steroidal anti-inflammatory drugs (NSAIDs) [8]. The cyclooxygenase (COX), target of NSAIDs is a key enzyme in synthesis of PGs, including PGE_2_, the main molecule acting on the thermoregulatory center to cause fever and pain [9]. PGE_2_ may also suppress host immunity by switching cytokines to a T helper-2 (Th2) phenotype [10]. Kubata *et al*. [11] demonstrated production of prostanoids in *P*. *falciparum* lysates and culture supernatants *in vitro*. *P*. *falciparum* also releases PGD_2_, E_2_, and F_2_α into the infecting milieu. The PG-synthesizing enzyme of *Plasmodium* has not been identified, but is resistant to NSAIDs including indomethacin and acetylsalicylic acid [11]. This finding suggests that the PG synthase of parasites evolved specifically to adapt the host immune response.

In this study, we identified production of SA by malaria parasites and investigated the role of SA *in vitro* and *in vivo*. We found that the PGE_2_ concentration is controlled by parasite SA *in vitro* and that induction of an SA-degrading enzyme in the parasite altered cytokine levels and increased mouse mortality *in vivo*. Based on these results, we propose a novel immunomodulatory pathway that includes parasitic SA and PGs.

## Materials and Methods

### Parasites and animals

*P*. *falciparum* strain 3D7 was provided by The Malaria Research and Reference Reagent Resource Center (MR4) and cultivated *in vitro* as described previously [12]. Human red blood cells (RBCs) and serum were provided by the Japan Red Cross. *P*. *berghei* strain ANKA was also provided by MR4. Passage, gene manipulation, and cloning of the parasite were performed as previously described [13] in ddY mice purchased from Japan SLC (Shizuoka, Japan). We passaged the parasite when parasitemia reached approximately 5%. One drop of blood (~5 μL) was suspended in 100 μL of phosphate-buffered saline (PBS) and injected intraperitoneally into another mouse. Parasite specimens for mass spectrometry analysis of SA were collected from whole blood of infected ddY mice using the same method of infection. All other experiments, including survival, experimental cerebral malaria (ECM) assessment, and cytokine and prostaglandin measurements were performed in the C57BL/6 mouse strain. Female mice aged 6–9 weeks old were purchased from Japan SLC. *T*. *gondii* strain RH and 2F were used with *in vitro* conditions [14]. *T*. *gondii* was cultivated with human foreskin fibroblast (HFF) cells (Millipore, Darmstadt, Germany). All animal experiments were conducted in accordance with the Guidelines for Animal Experimentation of the Japanese Association for Laboratory Animal Science and were approved by the Institutional Animal Care and Use Committee of the National Institute of Infectious Diseases (permission numbers: 112142, 213010, and 214004). All surgery was performed under isoflurane (Merck, Whitehouse Station, NJ) anesthesia and all efforts were made to minimize suffering.

### Salicylic acid determination

ddY mice were treated intraperitoneally with 50 μg/kg phenylhydrazine 1 day before infection to increase the ratio of reticulocytes and increase parasitemia. *P*. *berghei* ANKA was intraperitoneally injected into mice and parasitemia was checked daily. When parasitemia was >40%, whole blood was collected from the heart under deep anesthesia. The total parasitized RBCs were 1–1.7×10^9^ cells per sample. Blood parasites were purified by treatment with 1.5 mg/mL saponin in PBS to remove host contamination [15]. After three washes with PBS, the pellet was weighed and rapidly frozen in liquid nitrogen. Samples were stored at −80°C until use. *T*. *gondii* was collected as previously described [14]. Briefly, *T*. *gondii* tachyzoites were cultivated with HFF cells. Approximately 3×10^9^ parasites were purified by filtration with a 3-μm pore polycarbonate membrane. Few host cells contaminated the parasite sample based on observation under a microscope. The same procedure without *T*. *gondii* infection resulted in almost no weight gain of harvested cells (data not shown). After purification, parasites were washed three times with Hank’s balanced salt solution supplemented with HEPES buffer (10 mM final) and ethylene glycol tetra-acetic acid (EGTA, 1 mM final) and immediately frozen as above.

Extraction of SA was performed as described elsewhere [16,17]. Briefly, 1 ml of prechilled (-30°C) extraction solvent (methanol: formic acid: water, 15:1:4) and internal standards were added to each parasite pellet and the mixture was kept at -30°C for at least 16 h. After centrifugation at 10,000×*g* for 15 min, the supernatant was transferred to a 96-well collection plate (Waters, Milford, MA) that was placed on an automated solid phase extraction system (SPE215; Gilson, Middleton, WI). SA purification was performed by passing the extract through an Oasis HLB 96-Well Plate (30 mg; Waters) equilibrated with 1 M formic acid, followed by washing with 0.3 ml of extraction solvent. The SA-containing fraction was then passed through an Oasis MCX 96-Well Plate (30 mg; Waters) equilibrated with 1 M formic acid. After washing with 1 M formic acid, SA was eluted with 1 ml methanol. The SA fractions were then evaporated and reconstituted with 1 ml of water. Finally, 50 μl of the SA fraction was combined with 1 μl of 5% formic acid for liquid chromatography (LC)-mass spectrometry analysis.

The mass spectrometry-based identification was performed with a Nexera UHPLC/HPLC (Shimadzu)/Triple TOF 5600 (AB SCIEX) system with a ZORBAX Eclipse XDB-C18 column (Agilent). Elution condition of LC is described previously [16].

Quantification of purified SA was performed with an ultra-performance liquid chromatography (UPLC)-tandem mass spectrometry (Aquity UPLC System/XEVO-TQS; Waters, Milford, MA) with an ODS column (Aquity UPLC BEH C18, 1.7 μm, 2.1×50.0 mm; Waters) [16,17]. Quantification of SA from *P*. *falciparum* was performed using a commercial salicylate diagnosis kit (Roche, Basel, Switzerland) with direct use of culture supernatants.

### Addition of salicylic acid to a parasite culture *in vitro*

*P*. *falciparum* strain 3D7 and *T*. *gondii* strain 2F were cultured with media containing SA *in vitro*. After synchronization twice with 5% (w/v) D-sorbitol in Milli-Q water (Merck KGaA, Darmstadt, Germany) [11], *P*. *falciparum* was treated with 100 μM SA. Parasitemia was observed every 24 h. *T*. *gondii* 2F, a bacterial β-galactosidase (β-gal) expressing clone with a RH background, was allowed to infect HFF cells and immediately treated with 10^−1^ to 10^5^ nM SA. After cultivation for 48 h, parasite growth was quantified based on β-gal activity [14].

### Establishment of salicylic acid-deficient parasites

*P*. *falciparum* was transfected with the SA-degrading gene, *nahG*, using an episomal expression system [18]. Briefly, *nahG* was driven by *P*. *falciparum* chloroquine-resistant transporter gene 5′UTR (pCRT) and a *P*. *berghei* dihydrofolate reductase gene 3′UTR (PbDT) fused into an attR4-attR3 site of the destination vector pCHD43 (II) [19]. The constructed pCRT::nahG-cmyc vector was transfected by electroporation (S1 Fig). Parasites were selected with 5 nM WR99210 (a gift from Jacobus Pharmaceuticals). For *P*. *berghei* ANKA, the *nahG* expressing vector was constructed with the pL0006 vector (distributed from MR4) and *nahG* was also driven by pCRT and PbDT (S2 Fig). The vector was linearized and recombined into the *230p* gene locus, which has no known function [20]. The *P*. *berghei* ANKA transfectants were selected with 70 μg/mL pyrimethamine in drinking water (MP Biomedicals, Solon, OH) and cloned by limiting dilution [13]. Control parasites transfected with *gfp* were made in the same way. Expression of *nahG* was confirmed by Western blotting with rabbit anti-c-Myc polyclonal antibody (1:100 dilution) (Santa Cruz Biotechnology, Santa Cruz, CA), using the mouse anti-HSP-90 monoclonal antibody as control (Sigma Aldrich, St. Louis, MO). The goat anti-rabbit secondary antibody HRP conjugate (1:1,000) (Sigma Aldrich) was used for c-myc and the goat anti-mouse secondary antibody HRP conjugate (1:5,000) (Molecular Probes, Eugene, OR) was used for HSP-90. Signals were identified with SuperSignal West Pico Chemiluminescent Substrate (Thermo Scientific, Waltham, MA).

### Mice mortality assay

C57BL/6 mice received 10^4^ parasitized red blood cells intravenously. Survival, weight, parasitemia, and clinical signs were checked twice a day, and survival was recorded daily to prepare survival curves. Moribund mice in a coma, with no movement, no body extension and no response to any stimulus were euthanized by deep isoflurane anesthesia. The decision to apply euthanasia followed previous criteria [21]. Brain hemorrhage was examined as a potential cause of death. At 21 days after infection, surviving mice were sacrificed to minimize pain using the same method of euthanization.

### Evaluation of ECM

To evaluate disruption of the blood brain barrier (BBB), a dye leakage test was performed [22]. Briefly, mice at 6 days post-infection were intravenously injected with 100 μL of Evans blue dye (1% w/v in PBS; Sigma Aldrich). After 1 h, mice were anesthetized and euthanized to collect whole brains. The brains were photographed, weighed, and transferred into 2 mL formamide (Wako Chemicals, Osaka, Japan). Samples were incubated for 48 h at 37°C and dye extravasation was determined by measurement of optical density (OD) at 640 nm. Values were normalized using tissue weight. To evaluate sequestration of vessels in brains, samples were observed histologically. Mice 6 days post-infection were anesthetized, perfused through the heart with 5 mL of PBS, and then with 5 mL of ice-cold phosphate-buffered 4% paraformaldehyde solution (PFA; Wako Chemicals) for fixation. Brains were transferred into 4% PFA and fixed overnight at 4°C. Brain slices stained with hematoxylin and eosin were observed by microscopy.

### Prostaglandin and cytokine quantification

PGE_2_ was measured using a Prostaglandin E_2_ EIA Kit Monoclonal (Oxford Biochemical Research, Oxford, MI). Culture supernatant and plasma from heparin-treated whole blood were used for measurements. Mouse plasma samples from animals at 6 days post-infection were collected as above and cytokines were analyzed using a Bio-Plex Pro Mouse Cytokine 23-plex Assay and Th1/Th2 Assay Kit (Bio-Rad, Hercules, CA).

### Statistics

All statistical tests were performed using R software (ver. 3.0.0; R Foundation for Statistical Computing, Vienna, Austria [http://www.R-project.org/]). A Mann-Whitney U-test was used for non-parametric comparison of Evans blue leakage, plasma prostaglandins, cytokine quantification, and clinical signs (weight, parasitemia, and hematocrit). A log-rank test was used to compare survival curves of infected mice. A non-paired two-tailed Student t-test was used to compare *in vitro P*. *falciparum* results. Kaplan-Meier survival curves of parasite-challenged mice and box graphs of *in vivo* results were depicted using R software.

## Results

### Identification of salicylic acid in apicomplexan parasites

**Fig 1:**
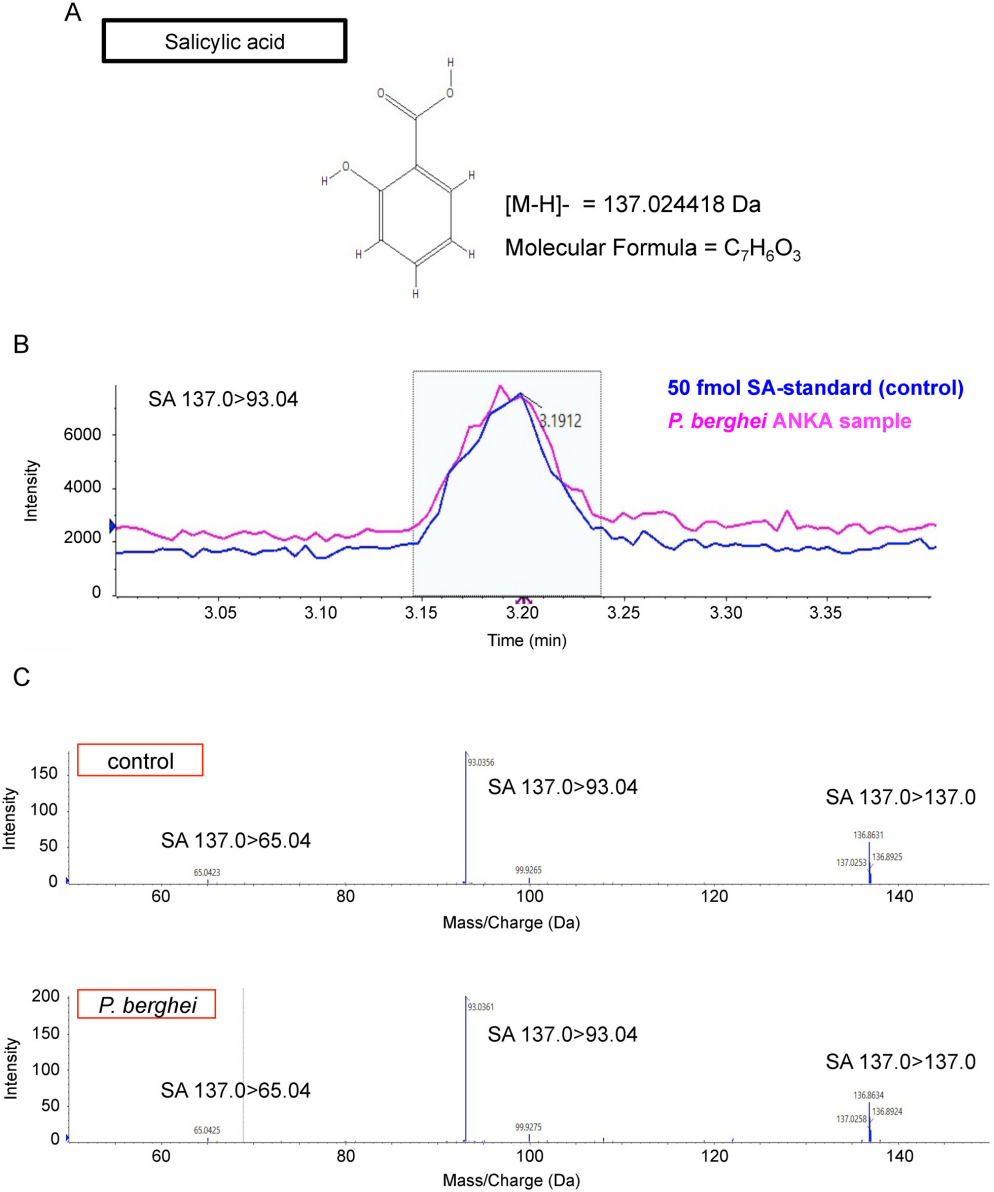
Identification of salicylic acid from *Plasmodium berghei* ANKA. *P*. *berghei* ANKA was purified from infected mice blood, and salicylic acid (SA) was extracted, and analyzed by LC-triple TOF mass spectrometry. (A) Structural formula of SA. (B) LC chromatogram of SA standard (control) and *P*. *berghei* ANKA sample. (C) Fragmentation analysis of peaks in (B) (colored in aqua). Collision energy was 20 eV.

**Fig 2:**
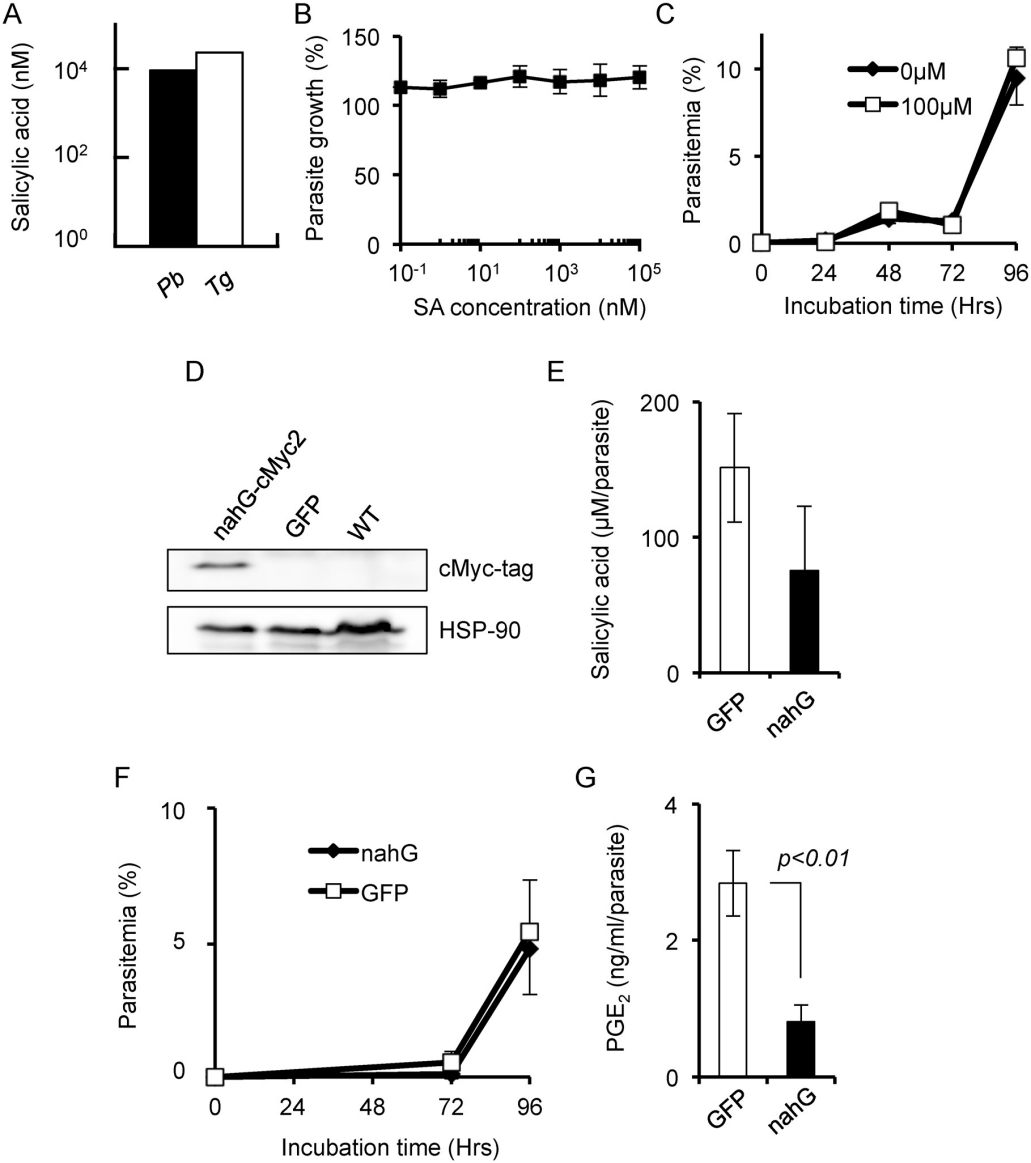
Quantification, *in vitro* function and genetic manipulation of salicylic acid (SA). (A) SA concentrations identified by automated quantitative mass spectrometry. Black and white bars show the SA concentrations in fresh cell lysates of *Plasmodium berghei* ANKA (Pb) purified from infected mouse blood *in vivo*, and *Toxoplasma gondii* RH (Tg) cultured *in vitro*, respectively. The concentrations were calculated based on the weight of parasite lysate. (B) Effect of addition of SA to culture medium on growth of *T*. *gondii* 2F. Growth was quantified by β-galactosidase activity after cultivation for 48 h. Each value was normalized using control parasites treated with DMSO. The graph shows the mean of two independent experiments. (C) Effect of addition of SA to culture medium on growth of *P*. *falciparum*. The growth rate was monitored by counting parasitemia by Giemsa staining. (D) Western blotting of *nahG* fused with a cmyc2-tag. HSP-90 was used as an internal control (bottom panel). (E–G) Biological changes of *P*. *falciparum* due to SA deficiency *in vitro*. (E) Secretion of SA into the culture supernatant by *P*. *falciparum* transfected with nahG-cmyc2 or GFP (control). (F) Growth effect of intracellular SA depression. Growth kinetics of *nahG*-containing mutants were compared with the control (*gfp*-transfectant). (G) SA affected the synthesis of prostaglandin E_2_ (PGE_2_) in parasites (p<0.01, Student t-test). All experiments with *P*. *falciparum* were performed after synchronization twice with 5% (w/v) D-sorbitol solution (n = 3). Bars indicate the data range (B) or standard error (C, E-G).

SA was extracted from *P*. *berghei* ANKA and analyzed by a LC-triple TOF mass spectrometry. Identical fragments to those for standard SA were detected in the parasite lysate, indicating that SA is present in this organism (Fig 1). For quantification, SA was extracted from *P*. *berghei* ANKA and *T*. *gondii*, respectively, and analyzed by an automated MS quantification system. Both parasites contained SA at remarkably high concentrations (*P*. *berghei*: 9.18 μM; *T*. *gondii*: 23.6 μM, respectively) (Fig 2A). Exogenous addition of SA did not alter parasite growth (Fig 2B and 2C), probably because the parasite already contained sufficient levels of SA.

### Establishment and analysis of SA-deficient parasites

Because addition of SA produced no significant phenotype, we transfected *P*. *falciparum* with the *nahG* gene, which encodes an SA-degrading enzyme found in the plant pathogenic bacteria *Pseudomonas putida* [23]. Expression of this gene product was confirmed by Western blotting using 2× myc-tag at the carboxyl-terminus of *nahG* (Fig 2D). The SA concentration of *nahG*-transfectants was lower than that in *gfp*-transfected controls, although the difference was not significant (Fig 2E). The growth kinetics and stage conversion of the parasites were similar (Fig 2F, S3 Fig). Taken together, these results indicate that the concentration of SA had no effect on parasite growth.

SA is an NSAID that modulates PGE_2_ levels in mammals. *Plasmodium* produces its own PGs, of which PGE_2_ is predicted to interfere with the immune system of a host [11]. We hypothesized that malaria parasites modulate the concentration of PGE_2_ in hosts through SA and their own production of PGE_2_. Therefore, we quantified the concentration of parasite prostaglandins. Surprisingly, the PGE_2_ concentration decreased significantly when the *nahG* gene was transfected into parasites (Fig 2G).

### Influence of *in vivo* SA deficiency on mouse survival

**Fig 3:**
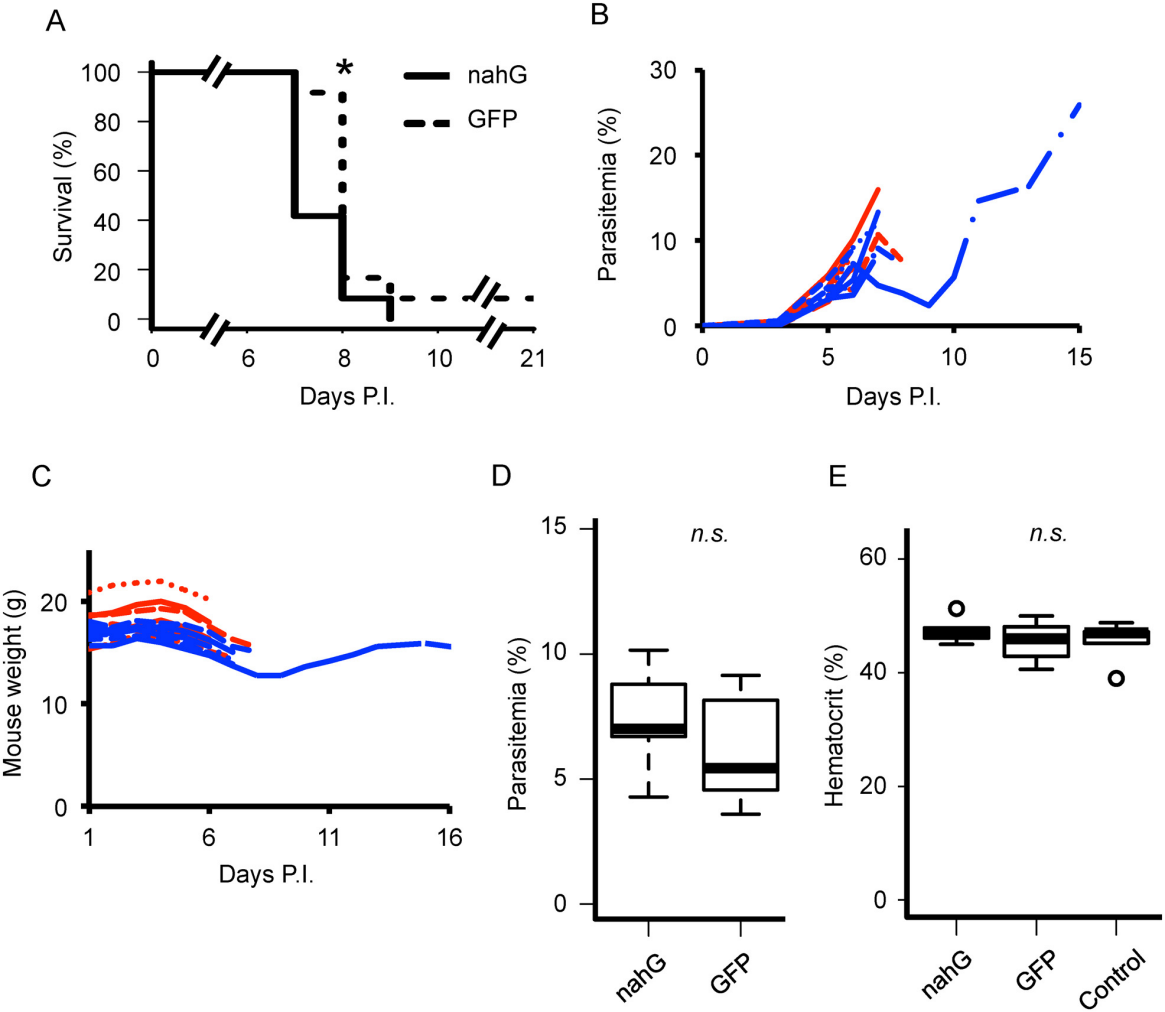
*In vivo* influence of SA depression. (A) Survival following infection of mice with *nahG*- or *gfp*-expressing parasites. Data are from two independent experiments, n = 13. *: p<0.05. (B, C) Parasite growth (B) and animal weight gain/loss (C). Each graph indicates an individual mouse infected by *nahG* (red) or *gfp* (blue)-expressing parasites. (D) Parasitemia of *nahG*- or *gfp*-expressing parasites at day 6 post-infection, when clinical signs were most significant. Plasma PGE_2_ and cytokines were quantified at this time point. (E) Hematocrit levels in mice at 6 days post-infection. Hematocrit did not differ between the groups, whereas the survival curves and clinical behavior shifted at the same time point. Statistics were calculated by Log-rank and general Wilcoxon test (A) and Mann-Whitney U-test (E).

To investigate the effect of SA *in vivo*, we established SA-deficient *P*. *berghei* ANKA by transfection with *nahG*. First, we examined the pathogenic effect of the SA deficiency. C57BL/6 mice were intravenously challenged with 10^4^ parasitized RBCs. As indicated in Fig 3A, mice challenged with *nahG* parasites had significantly increased mortality compared with the *gfp* parasite group (p<0.05, Log-rank test). We used three independent *nahG*-transfected clones and obtained similar results (data not shown). Both *nahG* and *gfp* parasites induced ECM, resulting in a neurological syndrome characterized by clinical signs including ataxia, reduced reflex, convulsions, and coma. Dissection of the heads of dead mice showed obvious brain hemorrhage. Thus, the final cause of death was concluded to be ECM for all mice in the *nahG* and *gfp* groups. However, onset of ECM was shortened by 1 or 2 days by infection with the *nahG*-transfectant compared with control parasites. One mouse survived for 21 days after infection and was then sacrificed to minimize the pain.

### Influence of *in vivo* SA deficiency on ECM

**Fig 4:**
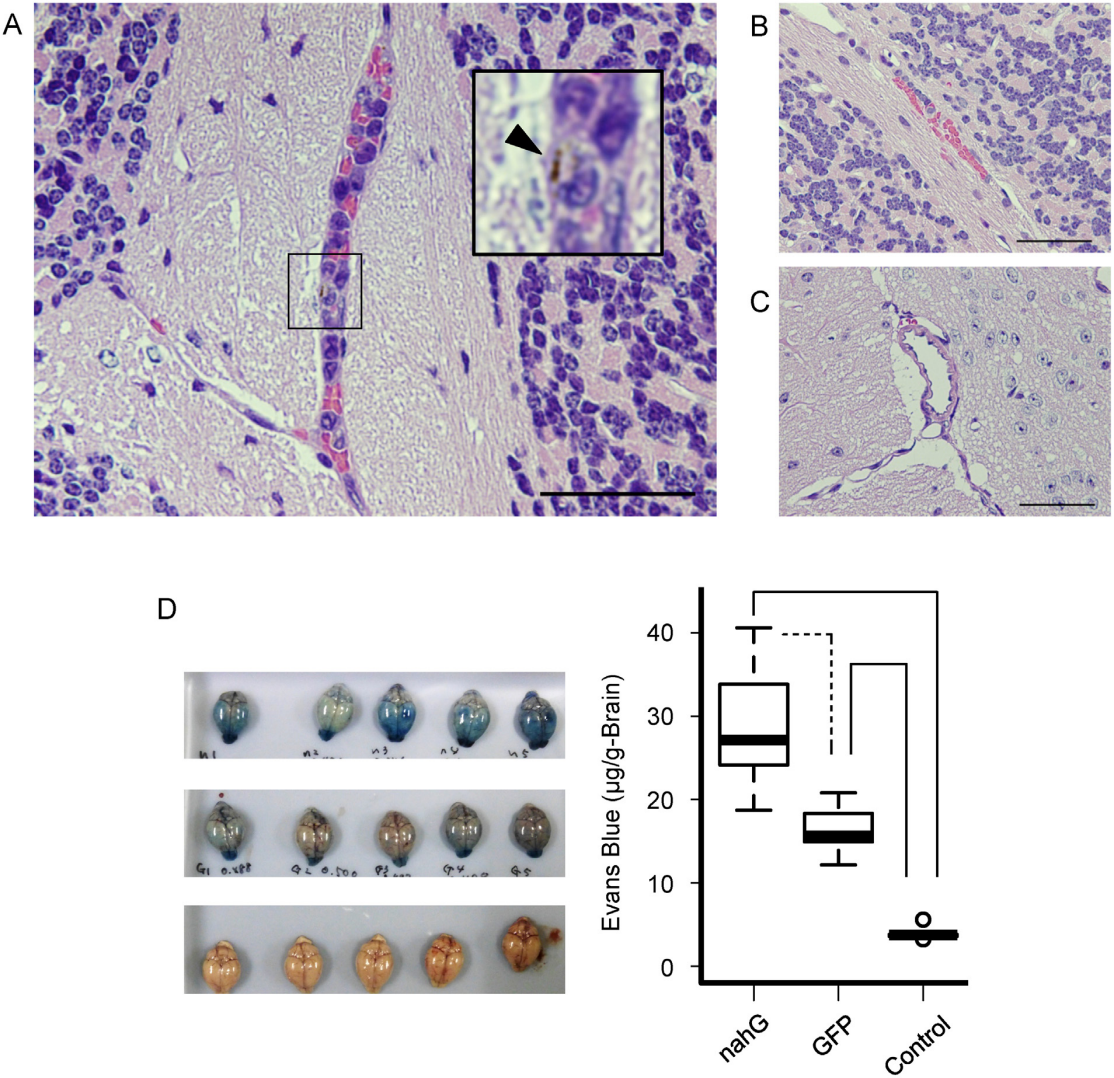
Parasite SA influences the cerebral malaria outcome. (A-C) Histological observation of infected mouse cerebellums. (A) Cerebellum infected by *nahG*-expressing parasites. Note the sequestrated leukocytes in microvessels. The inset image shows a higher magnification of the boxed portion. Phagocytized hemozoin is observed (arrowhead). (B) Brain of a mouse infected with *gfp*-expressing parasites. Slight microbleeding was observed, but no sequestrated vessels were found. (C) Brain of an uninfected control. Sections were stained by hematoxylin and eosin. (D) Evans blue leakage analysis of the severity of cerebral malaria. Photographs of brains from mice infected with *nahG*- (left upper) and *gfp-* (left middle) expressing parasites and uninfected controls (left bottom), and quantification of dye leakage (right). Mice (n = 5) were sacrificed at 6 days post-infection. Solid line, p<0.01; dashed line, p<0.05. C57BL/6 mice at 6 days post-infection were used for all experiments. Bar: 50 μm.

Since there seemed to be a difference in severity of ECM between mice with *nahG*- or *gfp*-expressing parasites, we analyzed the brains of infected mice histologically to evaluate ECM, including sequestration of microvessels and hemorrhage (Fig 4). The brain was stained with hematoxylin and eosin and the pathology was observed. The cerebellum of mice infected with *nahG* transfectants showed leucocyte sequestration in small vessels (Fig 4A). This pathological observation is a typical symptom of ECM [24]. Mice infected with *gfp* transfectants showed slight hemorrhage, but no sequestration of leukocytes, suggesting less severe ECM (Fig 4B). There were no pathological changes in vessels in uninfected control mice (Fig 4C). The midbrain, frontal cortex, occipital cortex and olfactory bulb had similar features (S4–S6 Figs). The sequestration of *nahG*-transfectants was significant compared to *gfp*-transfectants or uninfected control (S7 Fig): two of 3 *nahG*-transfectants showed significant differences, whereas one mouse (nahG-3) showed similar level of sequestration comparing to the *gfp-*transfectants (S7 Fig).

Next, we quantified the severity of ECM by Evans blue leakage assay (Fig 4D). Evans blue leakage was significantly higher in infected mice (p<0.01, *nahG* vs. control and *gfp* vs. control; p<0.05, *nahG* vs. *gfp*), indicating that *nahG* transfectants severely disrupted the BBB in ECM. Mouse parasitemia and body weight kinetics were also measured (Fig 3B and 3C). Parasitemia at day 6 after infection when mice infected with *nahG* transfectant but not *gfp* transfectant showed ECM signs, was not significantly different, suggesting that differences in the growth capacity of parasites did not explain differences in clinical changes (Fig 3D). Hematocrit scores on the same day were also equivalent (Fig 3E). Mouse weight did not differ and was even somewhat higher with *nahG* transfectant infection (Fig 3C). Thus, the differences in mortality may be explained by occurrence of ECM due to infection with *nahG* transfectants.

### Prostaglandins and cytokines in infected mouse plasma

**Fig 5:**
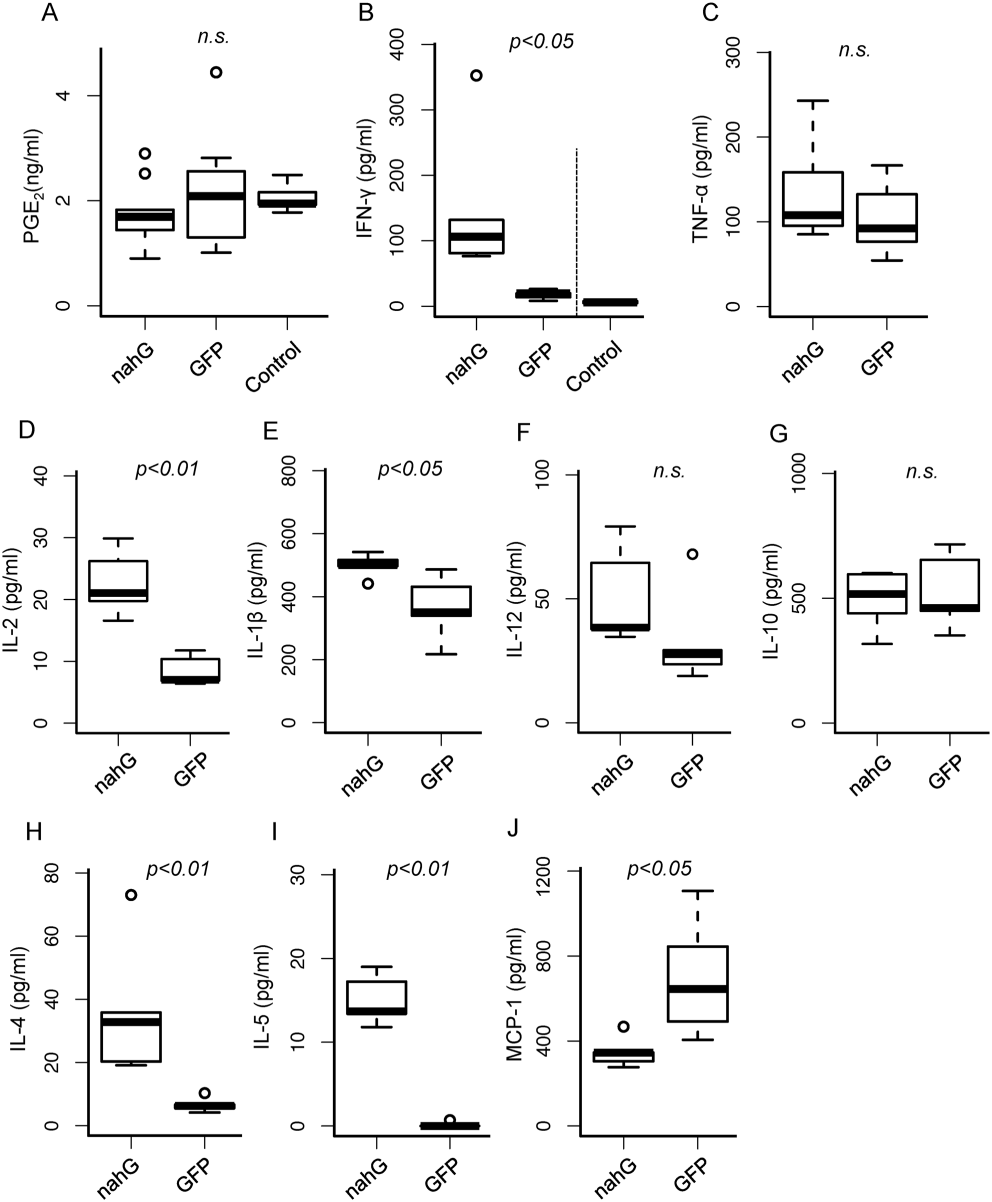
Plasma levels of cytokines, chemokines, and prostaglandin E_2_ (PGE_2_) in infected mice. (A) PGE_2_. (B-F) Proinflammatory cytokines: IFN-γ (B), TNF-α (C), IL-2 (D), IL-1β (E), and IL-12 (F). (G-I) Anti-inflammatory cytokines: IL-10 (G), IL-4 (H), and IL5 (I). (J) Inflammatory chemokine MCP-1. Plasma from heparin-treated whole blood of mice infected by *nahG*- or *gfp*-expressing parasites was used in all experiments. Control sample of IFN-γ was analyzed at the different day and indicated by dashed line. Statistics were calculated by Mann-Whitney U-test. Bonferroni correction was used for multiple tests.

The ECM evaluation showed enhanced pathogenicity of *nahG*-transfected parasites and the *in vitro* results indicated that a deficiency in SA affected production of PGE_2_ by the parasite. Therefore, we investigated the plasma concentrations of PGE_2_ and several cytokines influenced by PGE_2_. Whole blood (6 days post-infection) was collected for quantification of PGE_2_ and cytokines. We chose to detect the difference of PGE_2_ and cytokines on the sixth days post-inoculation because at this time point, only *nahG*-transfected parasites induced ECM. On the contrary, no change in parasitemia or hematocrit was detected as above (Fig 3B–3E), suggesting that the ECM could be induced by the differentially controlled PGE_2_ and cytokines. However, the plasma PGE_2_ level of mice infected with *nahG*-transfected parasites showed no decrease compared with *gfp*-transfectant mice or uninfected controls (Fig 5A), in contrast to the *in vitro* experiment.

The level of proinflammatory (T helper-1, Th1) cytokines in *nahG* transfectant-infected mice changed markedly. Interferon (IFN)-γ and interleukin (IL)-2 increased significantly (p < 0.05 and 0.01, Fig 5B and 5D) and IL-1β was also increased by infection with *nahG* parasites (Fig 5E, p < 0.05). Two other major proinflammatory cytokines, tumor necrosis factor (TNF)-α and IL-12, showed a similar shift, but the change was not significant (Fig 5C and 5F). The concentration of a T helper-2 (Th2) cytokine, IL-10, did not change significantly (Fig 5G), but IL-4 and IL-5 were significantly elevated in mice infected with *nahG* parasites (Fig 5H and 5I). MCP-1, an inflammatory chemokine, was significantly decreased by *nahG*-transfectant infection (p < 0.05, Fig 5J).

## Discussion

In this study, we found that *Plasmodium* spp. produces SA and may modulate the host immune response via PG production. The presence of SA was proven by mass spectrometry. Because we did not detect synthetic enzymes or receptors for SA in *Plasmodium* spp. genomes, we transfected *P*. *falciparum* and *P*. *berghei* ANKA with a SA-degrading gene, *nahG*, which was originally identified as a pathogenic factor in plant-infecting *Pseudomonas putida*. This gene encodes SA hydroxylase, which catalyzes SA to biologically inactive catechol [23]. *nahG* transfected *P*. *falciparum* had an approximately 50% decrease in SA concentration, which was a relatively small effect compared with results for plant transformants [23]. This might reflect differences in the intracellular environment (e.g., ions, pH, etc.) between land plants and *Plasmodium* spp. The SA concentration did not differ significantly at the 95% confidence level, but mutants of *P*. *falciparum* had significantly decreased endogenous production of PGE_2_, suggesting that the deficiency could be sufficient to explain the role of SA in *Plasmodium* spp. as a signaling molecule. Thus, production of PGE_2_ is probably dependent on SA in *P*. *falciparum*.

In animals, PGE_2_ usually functions as an active mediator of inflammation. It is released from whole cells, mainly vascular endothelial cells, fibroblasts and mast cells. Primary onset of fever is triggered through PGE_2_ receptor 3 expressed by neurons in the median preoptic nucleus [9]. PGE_2_ has complex activities in inflammation. It induces fever and pain, and has suppressive effects on both innate and acquired immune reactions. During innate immunity, PGE_2_ suppresses the functions and differentiation of natural killer cells, macrophages, granulocytes, and mast cells [10]. For example, the cytolytic activity of natural killer cells is suppressed by PGE_2_ through a mechanism involving suppression of IL-2, IL-12, and IL-15 [25,26]. Furthermore, macrophage functions are directly suppressed by PGE_2_ receptor 2-dependent signaling [27]. In acquired immunity, PGE_2_ inhibits production of IL-2 by T cells and IL-2 responsiveness [10]. It also suppresses T-cell activation and proliferation, and shifts the pattern of CD4^+^ T-cell responses from Th1 to Th2 and Th17 cells [10]. PGE_2_ directly prevents CD4^+^ T-cell production of IFN-γ, but not Th2 cytokines such as IL-4 and IL-5, in mice and humans [28,29]. Additionally, PGE_2_ prevents differentiation of CD4^+^ T-cell to Th1 by suppressing monocyte and dendritic cell production of IL-12 [10, 30]. Because PGE_2_ has such a complex role in immune activity, we hypothesized that the influence of a decrease in PGE_2_ in *nahG*-transfected parasites would lead to a distinct phenotype *in vivo*.

To investigate the function of parasite SA and induced PGE_2_, we established *nahG*-expressing *P*. *berghei* ANKA, and found that parasite virulence was significantly enhanced. The cause of death in infected mice was diagnosed as ECM based on the presence of neurological signs, clinical behavior, histological observation, and a dye leakage test. The severe pathogenicity caused by a malaria parasite is characterized by neurological signs in CM caused by sequestration of blood cells or disruption of the BBB in small vessels of the brain. Our result showed that microvessels of the brain, especially in the cerebellum, were sequestrated by many leucocytes and erythrocytes, and that the severity of sequestration was dependent on the transfected gene (*nahG* or *gfp*) (Fig 4, S4–S7 Figs). Disruption of the BBB was quantified by a dye leakage test, in which the significant difference in leakage indicated a difference in damage to the BBB. These results show that introduction of *nahG*, a SA-degrading enzyme, significantly enhanced the pathogenicity of *P*. *berghei* ANKA *in vivo*. However, we could not detect a significant difference in SA levels in *nahG*+ or *gfp*+ parasite infection, in contrast to the tendency for a decrease in SA level found *in vitro*. The *in vivo* environment may have influenced this finding due to egestion from the host or degradation by host metabolism, or the SA effect may have been locally restricted around the parasite.

Together with physical damage to brain vessels, an aggravating role of proinflammatory cytokines such as IFN-γ, TNF-α, and IL-12 in CM development is also widely accepted [31,32]. The mechanisms of CM development have been investigated by clinical observation of human patients and laboratory animal models of ECM. Elevation of serum IFN-γ is associated with the severity of acute malaria in Asian and African patients [33,34], and both *in vivo* IFN-γ neutralization and knock-out of IFN-γ receptor enhanced resistance against ECM caused by a rodent malarial parasite, *P*. *berghei* ANKA [35,36]. TNF-α also has a crucial role in the pathogenesis of CM since administration of an anti-TNF-α antibody completely protected against *P*. *berghei* ANKA infection [37]. Our study demonstrated that infection with the *nahG*-transfectant induced upregulation of proinflammatory cytokines including IFN-γ, IL-1β, IL-2, and IL-12, while parasitemia and hematocrit were almost uniform at the same time point (Fig 3D and 3E). This result suggests that these proinflammatory cytokines might play a role in aggravation of ECM with *nahG+* parasite infection.

The different elevation of proinflammatory cytokines suggests that the parasite actively modulates the host immunity. Taking into account the *in vitro* result, this modulation may occur via PGE_2_ produced by the parasite, which suppresses host immunity by switching cytokines to a T helper-2 phenotype [10]. Furthermore, PGE_2_ has a protective role in CM, with an inverse relationship between the concentration of plasma PGE_2_ and the severity of disease by *P*. *falciparum* infection found in a study in Gabonese children [38]. In a mouse model, the protective role of PGE_2_ was proven by administration of NSAIDs to infected mice [39,40]. These findings indicate the importance of PGE_2_ in protection against CM, and this effect can be explained by the suppressive activity of PGE_2_ on production of proinflammatory cytokines [10]. Therefore, elevation of proinflammatory cytokines due to the decrease of PGE_2_ in *nahG+* parasites may have resulted in enhancement of pathogenicity.

Another proinflammatory chemokine, MCP-1, underwent a decrease that differed from the changes in other proinflammatory cytokines. Generally, at the site of infection, MCP-1 is cooperatively elevated with other inflammatory substances and involved in the establishment of inflammation. In addition, release of MCP-1 from mast cells is promoted by PGE_2_ stimulation [41] and mast cells are thought to be the dominant source of MCP-1 [42]. Thus, the decrease of MCP-1 in *nahG+* parasite infection suggests that reduction of PGE_2_ production by the parasite may allow other cytokine levels to increase, but might reduce MCP-1 induction. Enhanced IL-4 and IL-5 in plasma from *nahG*-transfectant-infected mice is controversial because Th2 cytokines are generally protective against CM development, especially IL-4. However, a previous study showed that IL-4 was not associated with ECM pathogenesis [43]. Furthermore, few studies have focused on the role of IL-5 in severe malaria, including CM, and IL-5 production did not differ between ECM-susceptible and -resistant mouse strains [44]. The significant induction of IL-4 and IL-5 in the current study might reflect unknown cytokine functions in ECM development, which might be highlighted by *nahG+* parasite infection. Further analysis is needed to understand the roles of these cytokines in the pathogenesis of CM.

*Plasmodium* produces and releases PGs into the infecting milieu. In animals, the key enzyme in PG synthesis is COX, which catalyzes conversion of arachidonic acid to PGH_2_ [45]. Orthologs of COX have not been identified in the *Plasmodium* spp. genome *in silico*. In contrast to mammalian COX, the activity of which is inhibited by SA and other NSAIDs, an unidentified enzyme from *Plasmodium* spp. catalyzing the same reaction as COX was not inhibited by NSAIDs [11]. The current study showed that SA actually activates PGE_2_ synthesis, which suggests the presence of a novel signaling pathway. Additionally, we found that the change in plasma PGE_2_ levels was not significant in mutants of *P*. *berghei* ANKA *in vivo*, despite the *in vitro* result showing a significant decrease (p < 0.01). This suggests that the mechanism of PGE_2_ induction is different between *P*. *falciparum* and *P*. *berghei* ANKA, and the changes in mice mortality and ECM were caused by another mechanism. Otherwise, the difference of PGE_2_ level could be present only in the infection microenvironment. According to the previous report, the major source of IFN-γ in *P*. *berghei* ANKA infection is CD4^+^ T-cells at the site of infection [46]. Since PGE_2_ inhibits production of IFN-γ by CD4^+^ T-cells via direct and indirect ways [10,28–30], it is possible that the locally-concentrated PGE_2_ of parasite at the site of infection could influence the function of immigrating CD4^+^ T-cells. Investigation of this hypothesis will require analysis using a PGE_2_-receptor knock-out mouse.

SA was detected by quantitative UPLC-MS/MS for multi-phytohormone detection. This system allows identification of several plant hormones with high specificity [16,17]. The concentrations of SA in *P*. *berghei* ANKA and *T*. *gondii* were remarkably high compared with that in another phytohormone ABA (~200 nM) [3]. A high level of SA of almost 37 μg/g fresh weight is also found in the rice *Oryza sativa*, in which the concentration of SA is more than 1000-fold higher than that in the tobacco *Nicotiana tabacum* [47]. The high SA concentration in parasites is comparable and explains why there was no growth inhibition with exogenous addition of SA. In higher plants, such inhibition occurs when a compound is added at concentrations many times higher than the endogenous level. For example, the 90% inhibitory concentration (IC_90_) of ABA on *O*. *sativa* growth is 10 μM, which is almost 300-fold higher than the native level of 9.2 ng/g fresh weight (approximately 0.03 μM) [48].

SA has been studied for 100 years, but most studies have focused on anti-inflammatory effects in animals. In contrast, there have been few studies of signaling pathways and biosynthesis compared with other plant hormones [49]. The BA and ICS pathways have been identified as SA synthetic routes in higher plant, but the enzymes in these pathways have not been identified in any apicomplexan genome. Additionally, although NPR3 and NPR4 were recently identified as SA receptors [5], we could not identify the orthologs of NPR1, 3, or 4 in any apicomplexan genome database. This may be because of the diversity of higher plants and Apicomplexa or endosymbionts derived from early-branched red algae, as previously suggested for apicomplexan ABA synthesis [3].

In conclusion, a newly detected plant hormone SA may control endogenous PGE_2_ levels in *Plasmodium in vitro*. Our studies showed that introduction of *nahG* in *P*. *berghei* ANKA led to early onset of ECM *in vivo*. From our results, the most reasonable explanation is that this phenomenon is caused by overproduction of proinflammatory cytokines due to suppression of PGE_2_ production in parasites. This may be a new mechanism of *Plasmodium* spp.-induced modification of host immunity and this finding increases our understanding of the pathology of this threatening parasite.

## Supporting Information

S1 Fig Construction of a *nahG-cmyc2* expression vector for *Plasmodium falciparum* 3D7. (A) Promoter of *P*. *falciparum* chloroquine-resistant transporter gene (pCRT), terminator of *P*. *berghei* DHFR gene (PbDT), and *nahG-cmyc2* were amplified by PCR. (B) These fragments were joined by PCR with overlapping primers. The outermost primers contained 3′- and 5′- extensions that correspond to 20 bp sequences of the pCHD43 (II) vector, respectively. (C) The cassette of pCRT-nahG-cmyc2-PbDT was fused into pCHD43 (II) by homologous integration using a Geneart seamless cloning kit (Invitrogen) to give pCRT::nahG-cmyc2 (D).
(PDF)

S2 Fig Insertion of *nahG*-HA into the *Pb230p* locus in *P*. *berghei* genome. 500 bp sequences corresponding to the 5′ and 3′ regions of the *Pb230p* gene (which has no known function) were amplified by PCR and inserted into the pL0006 vector. Promoter of *P*. *falciparum* chloroquine-resistant transporter gene (pCRT), terminator of *P*. *berghei* DHFR gene (PbDT), and *nahG*-*HA* were amplified and joined by PCR with overlapping primers. The amplicon was also ligated into pL0006. For the control experiment, *gfp-HA* was amplified and introduced as for *nahG*. The constructed vector was cut by *Sac*II and *Not*I, and electroporated into *P*. *berghei* ANKA. Transfectants were selected by pyrimethamine. Expression was confirmed by western blotting with anti-HA antibody (lower panal).
(PDF)

S3 Fig Influence of *nahG* or *gfp* expression on the parasite development stage. *Plasmodium falciparum* 3D7 expressing *nahG* or *gfp* was established as shown in S1 Fig The parasites were cultured *in vitro*, synchronized twice with 5% sorbitol, and the stages were observed under microscopic observation. Parasites were examined at 72, 96 h after synchronization and the percentage was calculated based on 100 parasites per sample. There was no significant difference between the two transfectants. (p>0.05, Student T test, Bar: SD., n = 4).
(PDF)

S4 Fig Histological observation of mouse brain infected by *nahG*-HA expressing parasites. Brains of infected mice were perfused with PBS and fixed with 4% PFA. Sliced sections were stained by hematoxylin and eosin. The file includes pictures of the cerebellum, midbrain, frontal cortex, occipital cortex and olfactory bulb.
(TIF)

S5 Fig Histological observation of mouse brain infected by *gfp*-HA expressing parasites. Methods are same as S4 Fig.
(TIF)

S6 Fig Histological observation of uninfected mouse brain. Methods are same as S4 Fig.
(TIF)

S7 Fig Quantification of sequestrated brain vessels. Brains of infected or uninfected (control) mice were perfused with PBS and fixed with 4% PFA. Sliced sections were stained by hematoxylin and eosin. Cerebellums of the brains were photographed at the same magnification of S4–S6 Figs Five to 10 pictures per mice were counted (n>100 for each picture). A Mann-Whitney U-test with the Bonferroni's correction was used, and significant (p<0.05) differences were denoted by asterisks.
(PDF)
