# Supplementary figures and images for: Plant Hormone Salicylic Acid Produced by a Malaria Parasite Controls Host Immunity and Cerebral Malaria Outcome
Source: PLoS One. 2015 Oct 14;10(10):e0140559. doi: 10.1371/journal.pone.0140559 (PMC4605785; doi:10.1371/journal.pone.0140559)

# Supplementary Figure 1

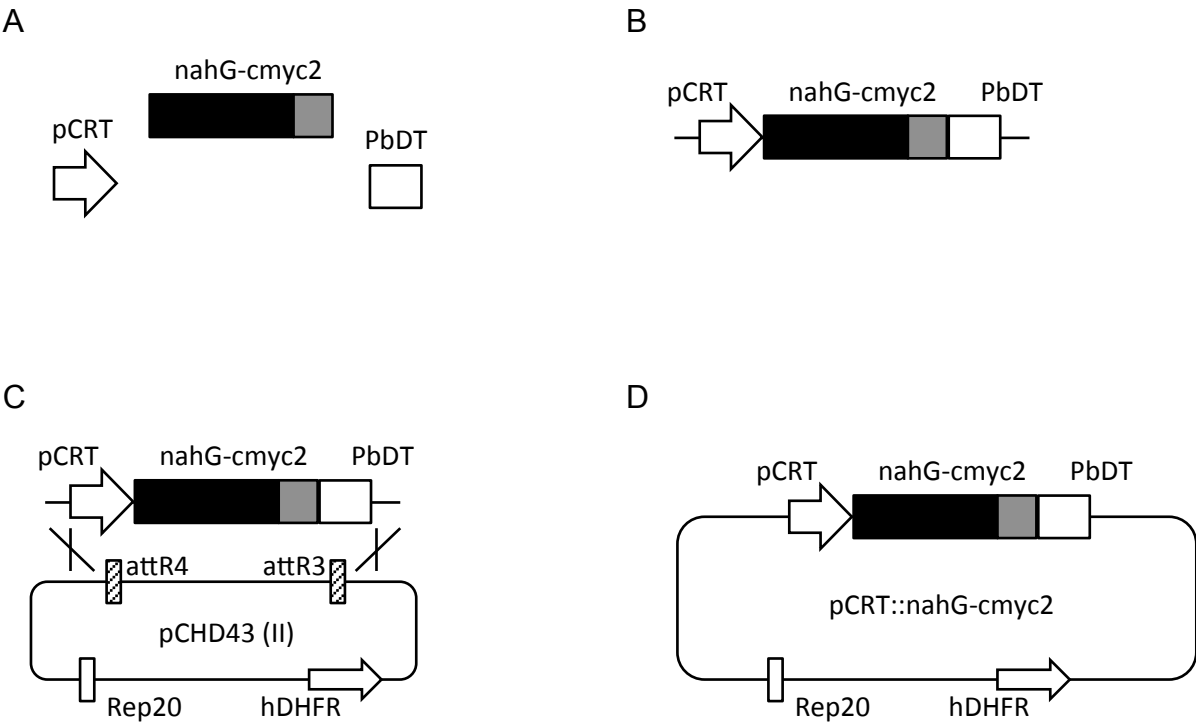

Supplement: S1 Fig — (A) Promoter of P. falciparum chloroquine-resistant transporter gene (pCRT), terminator of P. berghei DHFR gene (PbDT), and nahG-cmyc2 were amplified by PCR. (B) These fragments were joined by PCR with overlapping primers. The outermost primers contained 3′- and 5′- extensions that correspond to 20 bp sequences of the pCHD43 (II) vector, respectively. (C) The cassette of pCRT-nahG-cmyc2-PbDT was fused into pCHD43 (II) by homologous integration using a Geneart seamless cloning kit (Invitrogen) to give pCRT::nahG-cmyc2 (D). (PDF) [file pone.0140559.s001.pdf]

# Supplementary Figure 2

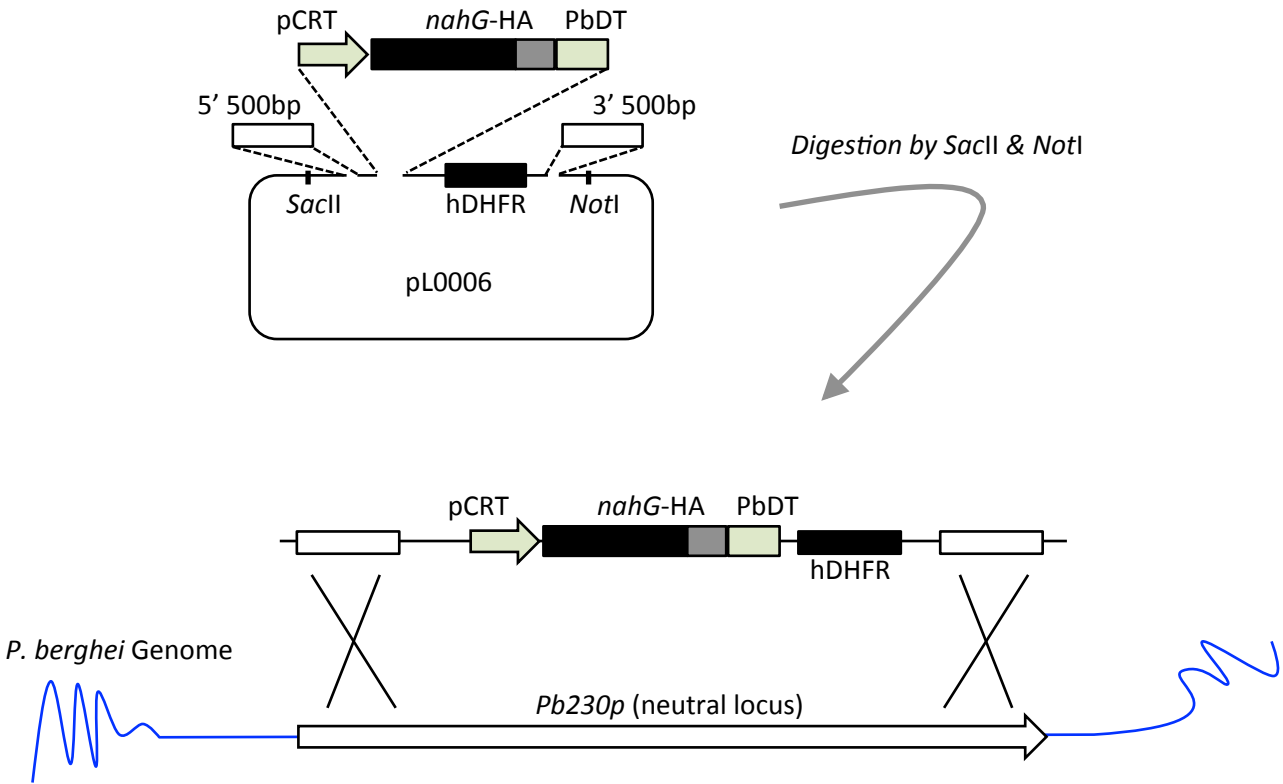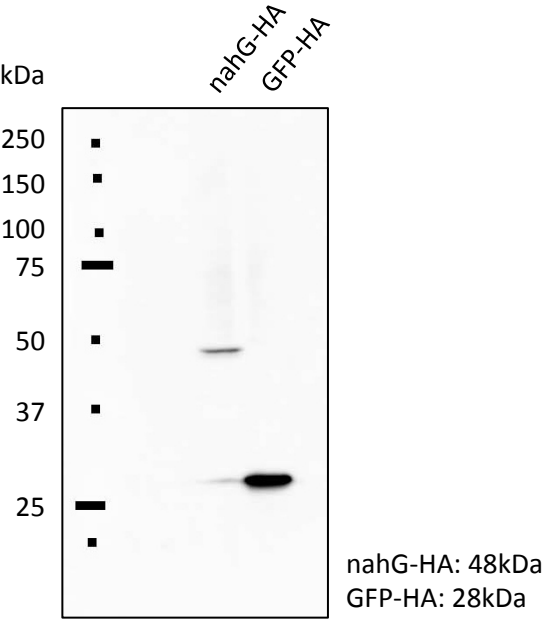

Supplement: S2 Fig — 500 bp sequences corresponding to the 5′ and 3′ regions of the Pb230p gene (which has no known function) were amplified by PCR and inserted into the pL0006 vector. Promoter of P. falciparum chloroquine-resistant transporter gene (pCRT), terminator of P. berghei DHFR gene (PbDT), and nahG-HA were amplified and joined by PCR with overlapping primers. The amplicon was also ligated into pL0006. For the control experiment, gfp-HA was amplified and introduced as for nahG. The constructed vector was cut by SacII and NotI, and electroporated into P. berghei ANKA. Transfectants were selected by pyrimethamine. Expression was confirmed by western blotting with anti-HA antibody (lower panal). (PDF) [file pone.0140559.s002.pdf]

Supplementary Figure 3

72 hours

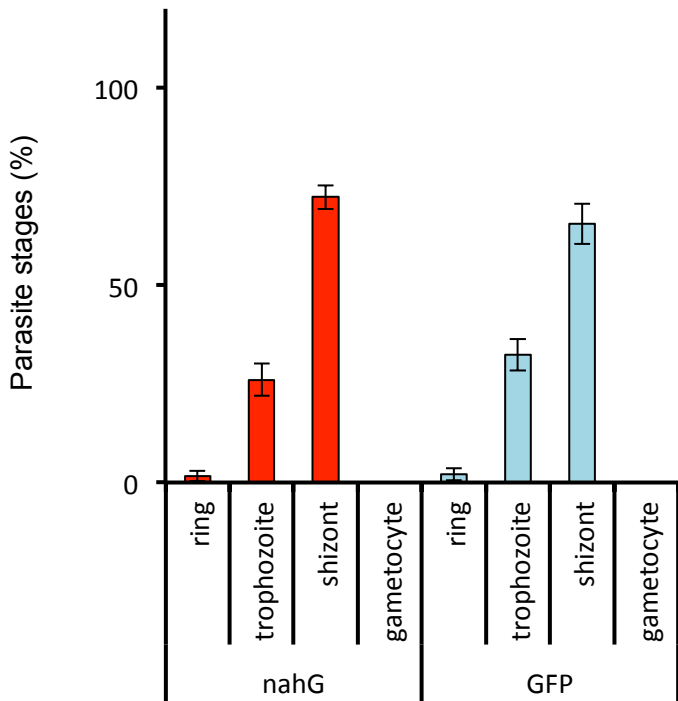

96 hours

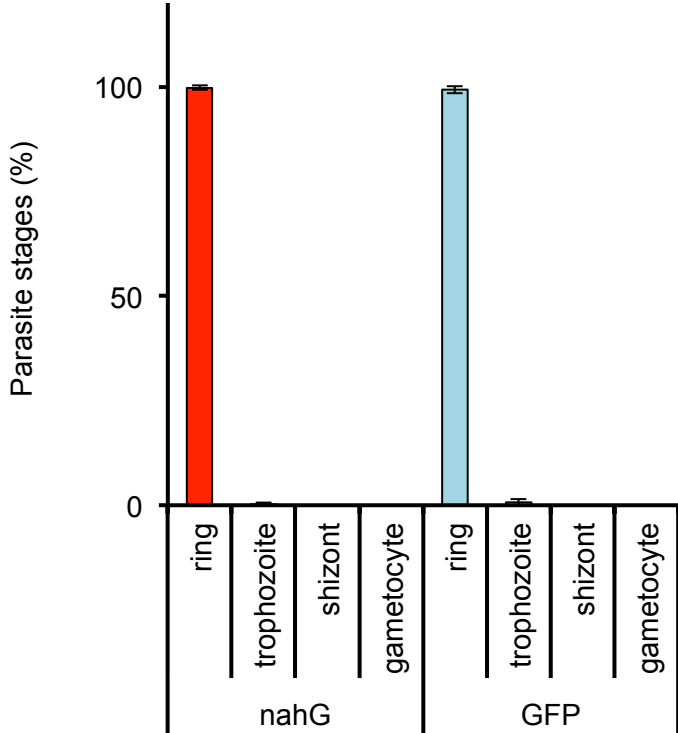

Supplement: S3 Fig — Plasmodium falciparum 3D7 expressing nahG or gfp was established as shown in S1 Fig The parasites were cultured in vitro, synchronized twice with 5% sorbitol, and the stages were observed under microscopic observation. Parasites were examined at 72, 96 h after synchronization and the percentage was calculated based on 100 parasites per sample. There was no significant difference between the two transfectants. (p>0.05, Student T test, Bar: SD., n = 4). (PDF) [file pone.0140559.s003.pdf]

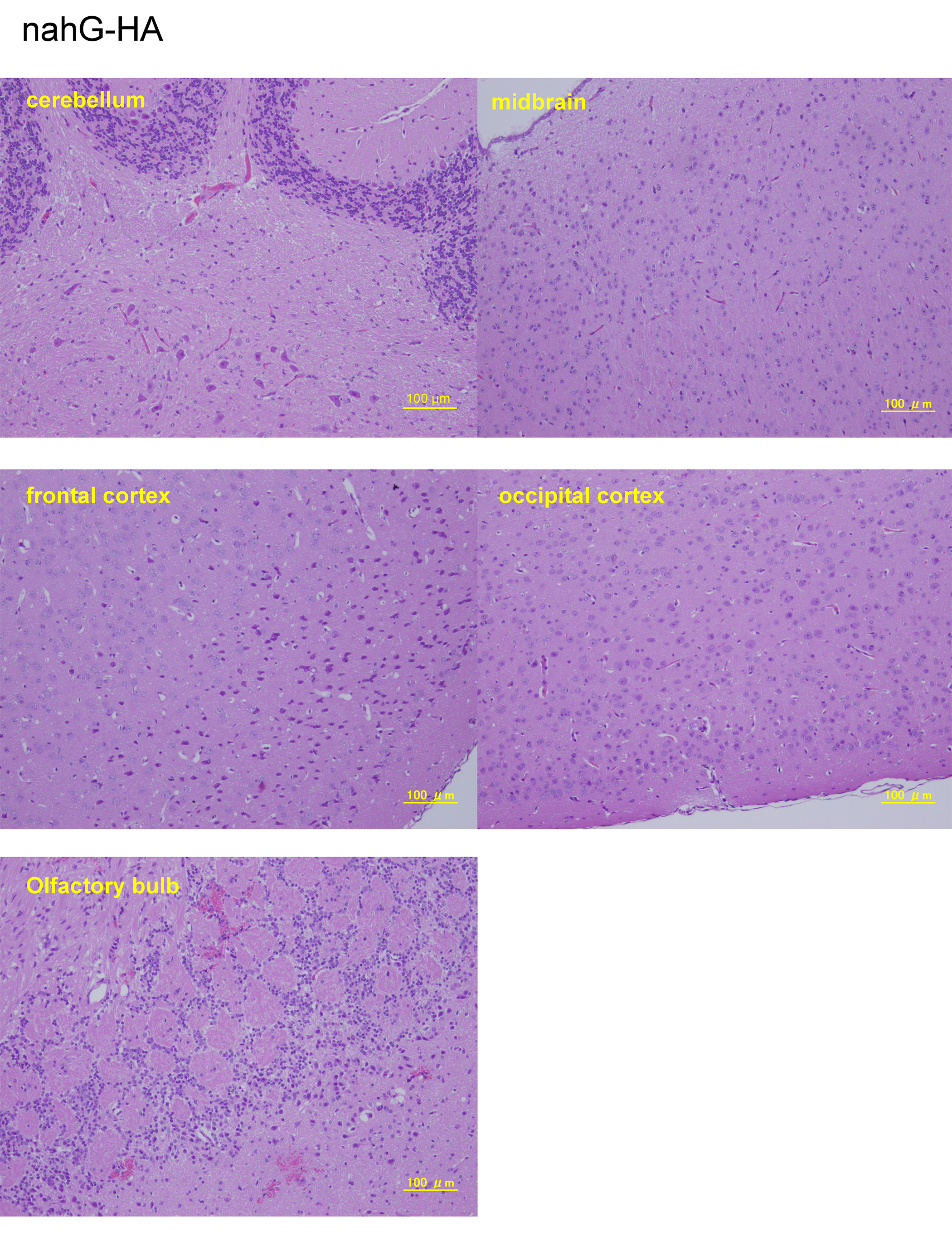

Supplement: S4 Fig — Brains of infected mice were perfused with PBS and fixed with 4% PFA. Sliced sections were stained by hematoxylin and eosin. The file includes pictures of the cerebellum, midbrain, frontal cortex, occipital cortex and olfactory bulb. (TIF) [file pone.0140559.s004.tif]

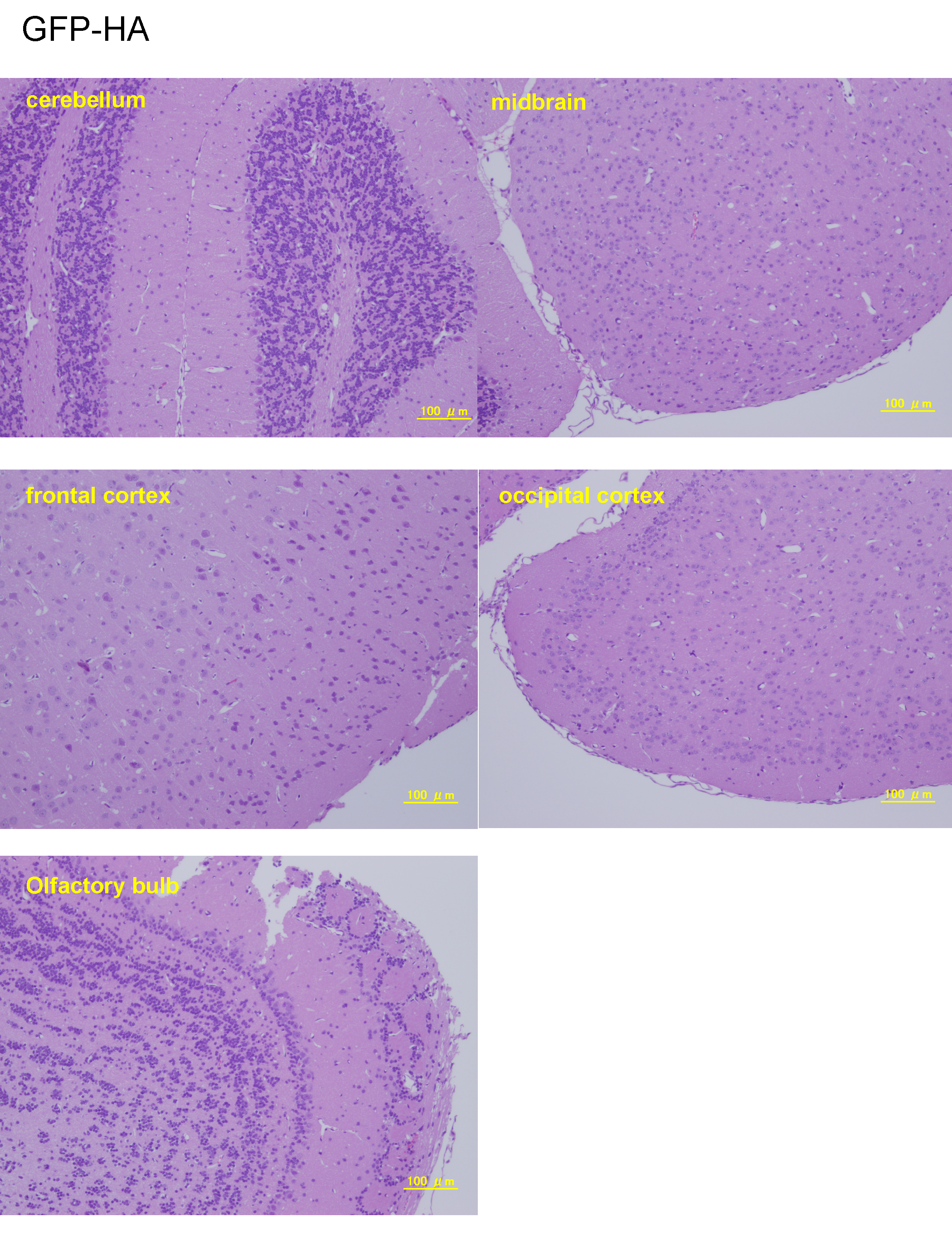

Supplement: S5 Fig — Methods are same as S4 Fig. (TIF) [file pone.0140559.s005.tif]

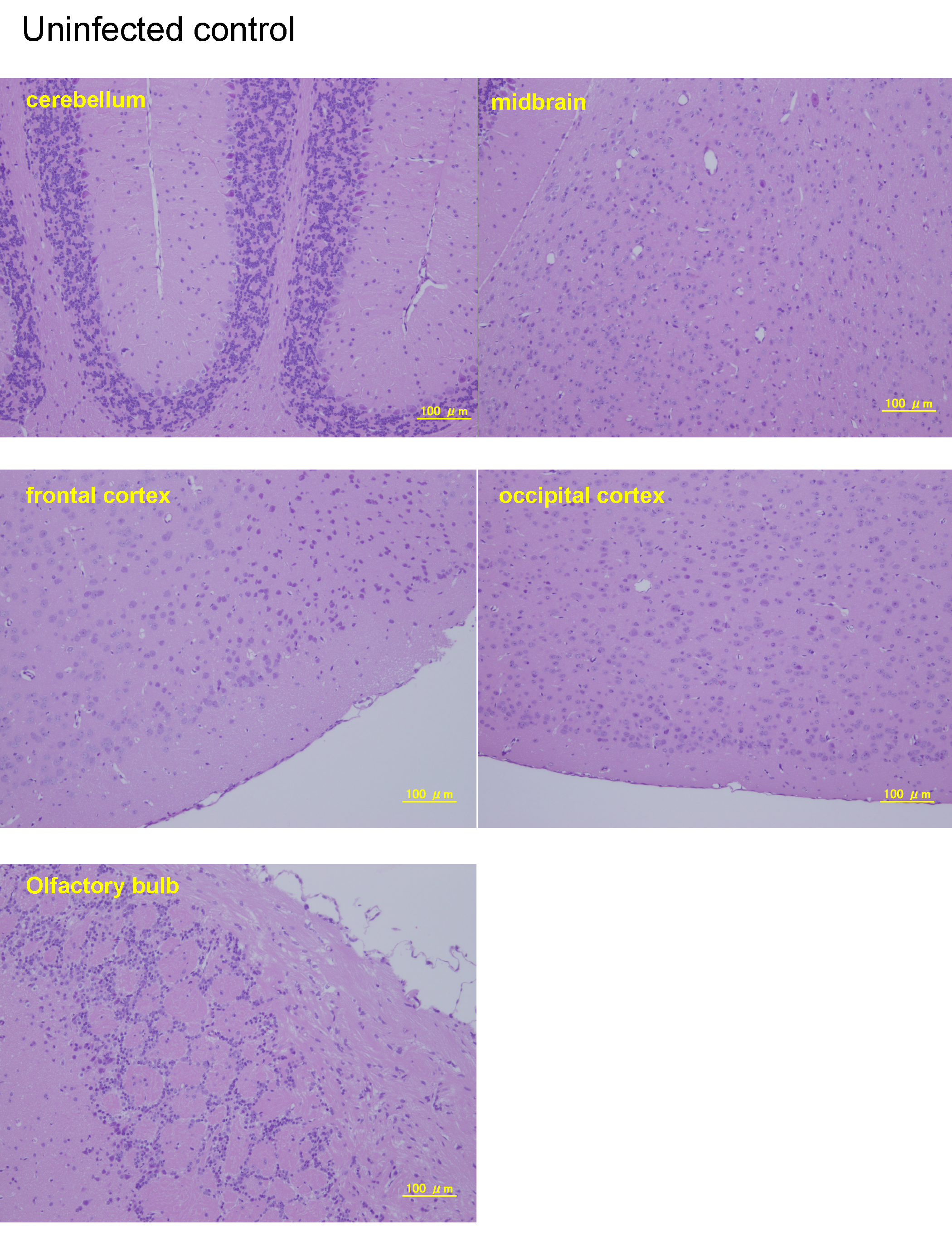

Supplement: S6 Fig — Methods are same as S4 Fig. (TIF) [file pone.0140559.s006.tif]

## Supplementary Figure 7

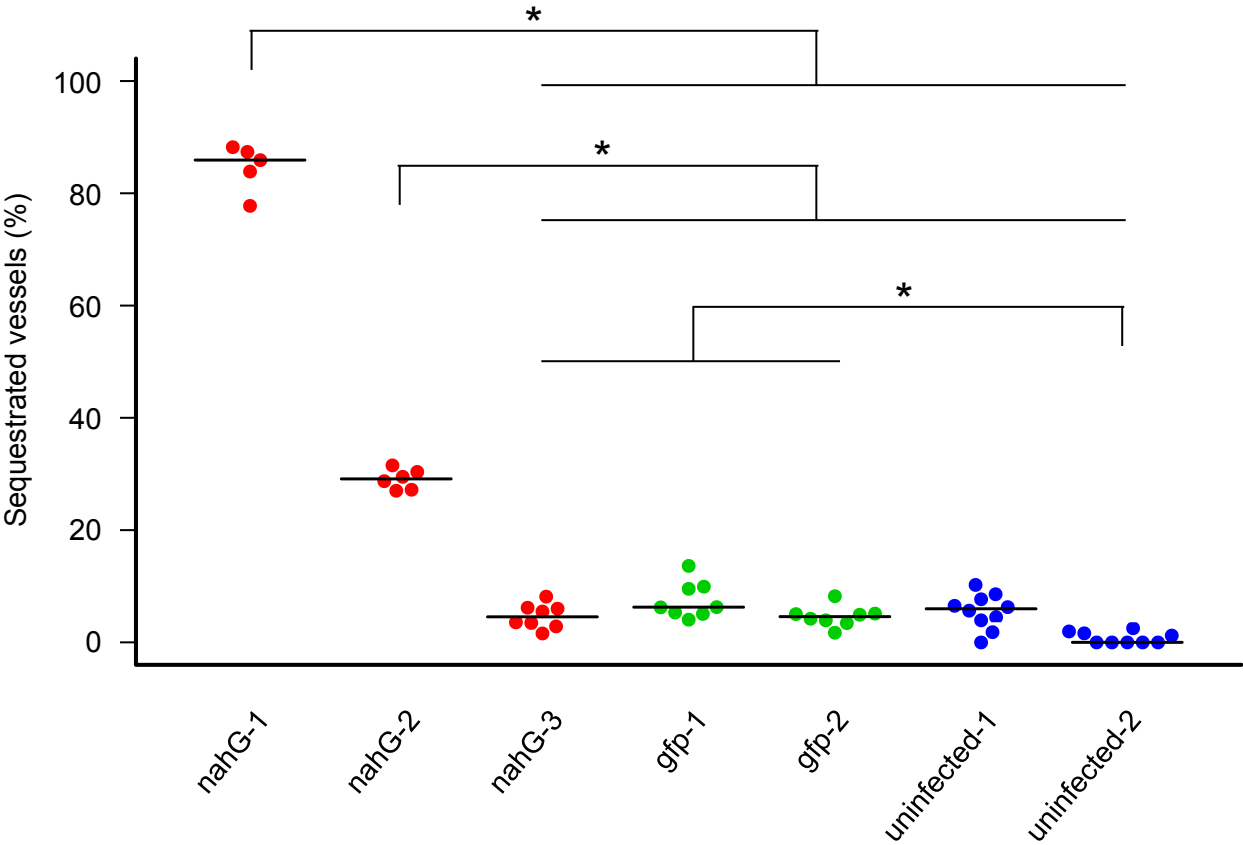

Supplement: S7 Fig — Brains of infected or uninfected (control) mice were perfused with PBS and fixed with 4% PFA. Sliced sections were stained by hematoxylin and eosin. Cerebellums of the brains were photographed at the same magnification of S4–S6 Figs Five to 10 pictures per mice were counted (n>100 for each picture). A Mann-Whitney U-test with the Bonferroni's correction was used, and significant (p<0.05) differences were denoted by asterisks. (PDF) [file pone.0140559.s007.pdf]
